# A Novel Image Encryption Scheme Based on Two-Dimensional Chaotic Map Constructed from Ackley Function and DNA Operations

**DOI:** 10.3390/e28030322

**Published:** 2026-03-13

**Authors:** Chao Jiang, Xiong Zhang, Xiaoqin Zhang

**Affiliations:** 1 College of Optical and Electronic Technology, China Jiliang University, Hangzhou 310018, China; silenceim007@gmail.com; 2 School of Optoelectronic Science and Engineering, University of Electronic Science and Technology of China, Chengdu 610054, China; 3 Department of Applied Physics, Nanjing University of Science and Technology, Nanjing 210094, China; 4 School of Information and Electronic Engineering (Sussex Artificial Intelligence Institute), Zhejiang Gongshang University, Hangzhou 310018, China

**Keywords:** chaotic map, image encryption, DNA encoding, DNA operation

## Abstract

In contemporary communication systems, digital images occupy an irreplaceable role; however, the privacy-related risks attendant to their prevalent application have grown increasingly salient. This paper presents an image encryption scheme integrating a novel two-dimensional Ackley-Sine chaotic map (2D-ASM) with dynamic DNA operations. First, a two-dimensional Ackley-Sine chaotic map, constructed based on the Ackley function and sine function, is designed and validated through a series of chaotic indicators. Results demonstrate that 2D-ASM exhibits superior chaotic properties compared to several existing state-of-the-art chaotic maps, with its maximum Lyapunov exponent (LE) exceeding 23, Permutation Entropy (PE) close to 1 in the full parameter range, and correlation dimension (CD) significantly higher than comparative chaotic systems. The proposed 2D-ASM-based image encryption scheme leverages the SHA-256 hash value of the plaintext image and four external keys to jointly generate the initial conditions and parameters of the 2D-ASM chaotic system, thereby ensuring a sufficiently large key space of 2^256^. Subsequently, chaotic sequences generated by 2D-ASM are employed to permute and diffuse the plaintext image, followed by dynamic DNA coding, operations, and decoding to obtain the encrypted image. Security analyses and comparisons with several existing representative algorithms confirm that the proposed encryption scheme achieves excellent encryption performance: the Number of Pixels Change Rate (NPCR) is above 99.6%, the Unified Average Changing Intensity (UACI) approaches 33.4%, and the information entropy of ciphertext images reaches 7.999 or higher. The scheme can effectively resist various potential attacks, including statistical and differential attacks, and outperforms representative algorithms in pixel correlation reduction and anti-interference performance.

## 1. Introduction

With the rapid advancement of modern communication technologies, digital images have emerged as a pivotal digital carrier for bearing and transmitting sensitive information, such as personal privacy, trade secrets, and military secrets. A vast volume of image data is generated, stored, replicated, and circulated across open-access environments, exposing such digital assets to substantial security vulnerabilities—including but not limited to surveillance, malicious tampering, unauthorized intrusion, and deliberate destruction. These threats may lead to the leakage of personal privacy, disrupt normal business operations, and even threaten national security. Consequently, developing effective strategies to safeguard the information security of digital images has become increasingly crucial [1].

To tackle the aforementioned security challenges, image encryption technology has emerged as a straightforward and highly effective approach. The fundamental concept of image encryption lies in converting an identifiable image containing valid information into an indistinguishable random noise-like image. Image encryption techniques are broadly classified into four categories, i.e., optical, spatial, transform domain, and compressive sensing [2]. Among the diverse array of image encryption techniques, chaos-based methods have garnered growing attention, owing to their distinctive advantages [3,4]. As inherently nonlinear dynamical systems, chaotic systems exhibit distinctive features, including ergodicity, pseudo-random behavior, unpredictability, and extreme sensitivity to initial conditions and control parameters [5]. These unique properties render them highly compatible with the cryptographic demands of critical operations, such as key generation, confusion, and diffusion [4]. Since researchers first introduced chaotic maps into the field of image encryption [6], chaotic systems have been widely utilized to develop numerous encryption schemes. Cao et al. [7] introduced a two-dimensional infinite collapse map (2D-ICM) and subsequently developed the ICMIE image encryption algorithm, which exhibits excellent hyperchaotic characteristics and ergodicity, and the algorithm has low computational complexity for pixel permutation. But the key space of the system is relatively limited, and the lack of association between the encryption process and plaintext features leads to insufficient resistance to chosen-plaintext attacks. Gao [8] constructed a 2D hyperchaotic system by integrating two 1D chaotic maps, a linear function, and a multiplier, and designed an encryption algorithm based on row–column permutation and forward–backward diffusion. This scheme adopts a simple and easy-to-implement permutation–diffusion structure. Nevertheless, the dynamic complexity of the chaotic system is insufficient, and the linear combination of 1D maps results in a narrow chaotic range and is prone to dynamical degradation under finite-precision computation. Hua et al. [9] proposed a Color Image Encryption Algorithm (CIEA) by combining Latin squares with a two-dimensional Latin square chaotic system (2D-LSM). This algorithm fully accounts for the channel correlation of color images and achieves effective confusion of multi-channel pixels. However, the Latin square operation involves high computational complexity, and the static encryption rules reduce the adaptability of the algorithm to various types of images. Teng et al. [10] presented a color image encryption scheme based on a cross 2D hyperchaotic system, combined with chaos-driven bit-level permutation and diffusion. Bit-level operations greatly enhance the encryption complexity. Unfortunately, the cross-structured chaotic map exhibits poor ergodicity in the low-parameter range, and the algorithm lacks an effective design against cropping attacks. Nan et al. [11] constructed a hyperchaotic system by integrating the Logistic map and the Cubic map, and combined it with a block compressive sensing algorithm to realize simultaneous encryption and compression for remote sensing images. This approach not only strengthens the resistance of images against various attacks but also significantly improves encryption efficiency. However, the compressive sensing procedure leads to irreversible loss of image information, and the encryption security degrades at low compression ratios. Zhu et al. [12] proposed a visually secure image encryption algorithm that integrates a two-dimensional fractional-order discrete chaotic map (FOCM), Bayesian compressive sensing (BCS), and the Discrete V Transform (DVT). This innovative algorithm greatly promotes both image security and compression performance, achieving a favorable balance between security protection and efficiency. Nevertheless, fractional-order operations impose high computational complexity, making the algorithm inapplicable to real-time encryption scenarios. Hu et al. [13] proposed a 3D hyperchaotic map with hidden attractors and an asymmetric semi-tensor product compressive sensing model. By combining them with Haar transform embedding in the orthogonal YCbCr color space, they established a visually secure image encryption algorithm that integrates compression, encryption, and carrier embedding. This scheme alleviates chaotic dynamic degradation and improves encryption security and efficiency, but it is still insufficient in resisting geometric attacks such as rotation and scaling. Yu et al. [14] proposed a non-polynomial memristor satisfying the Lipschitz condition, constructed three memristive Hopfield Neural Networks (HNNs) for different scenarios, verified the controllable generation of multiscroll attractors through dynamic analysis, adaptive synchronization design, and FPGA implementation, and developed an image encryption circuit based on the proposed network. Feng et al. [15] presented a 2D variable fractional-order coupled quadratic hyperchaotic map (2D-VFCQHM) with state-dependent dynamic memory, and developed a high-performance image encryption algorithm (IEA-VMFD) integrated with a dynamic vector-level diffusion-scrambling framework. Experimental results demonstrate that the algorithm achieves a good balance between security and efficiency and is suitable for practical applications. Du et al. [16] proposes an image encryption algorithm based on a hybrid 1D-2D cross-feedback hyperchaotic system and diffusive DNA coding, verifying its dynamic performance and security. It features a large key space, strong anti-attack ability and low complexity, but DNA coding is inefficient in simulations. Zhang et al. [17] proposes 2D-ELSCM hyperchaotic map and an image encryption scheme with IKDS and dynamic DNA-Zigzag encoding, using SHA-512 for key generation. It has strong chaos, robust attack resistance and large key space, yet suffers from fixed Zigzag paths and needs better key transmission security. These applications fully illustrate the considerable potential of chaotic systems in the field of secure communications.

Despite the potential demonstrated by chaos-based encryption methods, their core component—the chaotic map—still faces numerous challenges in practical applications, which directly impact the security strength and reliability of the final encryption scheme [18]. Firstly, many classic one-dimensional (1D) chaotic maps—such as the well-known Logistic map—though easy to implement, often exhibit insufficiently complex dynamics, characterized by narrow and discontinuous chaotic ranges interspersed with numerous non-chaotic “periodic windows” [19,20,21]. This not only limits the freedom of parameter selection but, more critically, allows minute parameter perturbations or rounding errors to potentially drive the system out of its chaotic state, resulting in a drastic drop in encryption performance [22]. Furthermore, such simple maps typically have few control parameters, directly leading to a relatively limited key space and increased vulnerability to brute-force attacks [23]. Secondly, a common issue inherent in various chaotic systems is dynamical degradation on finite-precision computing platforms [24,25]. Due to the precision limitations of computer representation and arithmetic, the long-term behavior of chaotic systems can deviate from theoretical infinite-precision trajectories, eventually settling into short periodic cycles—severely undermining the foundation of pseudo-random sequence generation [26]. Moreover, even with high-dimensional chaotic systems, encryption schemes may still struggle to effectively resist specific cryptanalytic attacks, such as chosen-plaintext attacks [27,28,29,30]. Concurrently, increased complexity often entails higher computational costs—a non-negligible factor in application scenarios demanding high-efficiency encryption. Thus, despite the wide variety of existing chaotic maps, achieving an optimal balance among robustness, resistance to degradation, security, dynamical complexity, and computational efficiency remains an ongoing challenge. This strongly motivates the cryptographic community to continuously explore and design novel high-performance chaotic systems better suited for encryption applications.

On the other hand, DNA (Deoxyribonucleic Acid) coding technology also plays a prominent role in the field of image encryption [31,32]. DNA molecules, characterized by their capacity for massive parallel processing, high information storage density, and unique base-pairing and operational rules [33,34], exhibit distinct advantages in cryptographic applications [35,36,37,38,39]. Integrating DNA operations into encryption algorithms not only significantly enhances the complexity of the encryption process but also leverages the diversity of DNA encoding rules to expand the key space [40], thereby potentially boosting the overall security of the encryption system [41]. For instance, DNA technology theoretically offers an effective solution to the storage and management challenges faced by the traditional One-Time Pad (OTP) in cryptography [42]: as high-density information carriers, DNA strands are well-suited for storing large-scale keys, a capability crucial for resisting powerful analytical methods such as chosen-plaintext attacks. Given the superiority of chaotic systems in generating pseudo-random sequences and the potential of DNA computing in parallel processing and information encoding, combining high-performance chaotic systems with DNA encoding and operational rules has emerged as a highly active and promising research direction in image encryption in recent years [43]. This integration aims to utilize chaotic systems for efficient pixel permutation and diffusion, while leveraging DNA-level operations to further enhance confusion effects and algorithmic complexity, thereby constructing more secure and reliable image encryption schemes [26]. However, it should be noted that despite incorporating DNA technology, some existing chaos-DNA encryption schemes still suffer from security flaws. For example, the DNA encoding/decoding rules in certain schemes are static [44,45,46], or their encryption processes (including key generation or DNA rule selection) lack correlation with the plaintext image content—rendering them potentially vulnerable to known-plaintext or chosen-plaintext attacks [47]. Therefore, designing encryption algorithms that can more tightly and securely integrate chaotic dynamics with dynamic, plaintext-associated DNA operations remains a critical issue worthy of in-depth exploration.

In light of the above context, this paper is dedicated to designing a high-performance novel chaotic system and exploring secure integration approaches with dynamic DNA operations. To this end, we first propose a novel two-dimensional chaotic map, designated as the 2D Ackley-Sine Map (2D-ASM). Leveraging this 2D-ASM chaotic map, we further develop and implement a novel image encryption algorithm. The core idea of this algorithm lies in the deep integration of chaotic sequences generated by 2D-ASM with dynamic DNA sequence operations that are closely associated with plaintext information. Specifically, we not only utilize 2D-ASM for efficient pixel permutation and diffusion but also dynamically regulate the DNA encoding and operation processes by integrating chaotic sequences with plaintext features, thereby ensuring high sensitivity of the encryption process to the plaintext. This design overcomes the limitation of traditional chaos-DNA schemes where keystreams or DNA rules are decoupled from the plaintext, significantly enhancing the algorithm’s resistance to known-plaintext and chosen-plaintext attacks. Through the synergistic effect of 2D-ASM’s superior chaotic properties and the complexity of dynamic DNA operations, we ultimately achieve a highly secure, efficient, and robust image encryption scheme.

The main contributions and innovations of this paper are summarized as follows:

(a) Proposing and validating a novel two-dimensional chaotic map (2D-ASM): A new 2D-ASM chaotic map is designed, and its superior chaotic performance—including a wider chaotic range, enhanced ergodicity, and more complex dynamics—compared to several existing maps is demonstrated through multi-dimensional analyses, such as bifurcation diagrams, Lyapunov exponent, information entropy, correlation dimension, and NIST randomness tests.

(b) Integrating chaos and DNA in the encryption algorithm: 2D-ASM chaotic sequences are dynamically incorporated into DNA encoding, operations, and decoding processes to support the permutation and diffusion stages of image encryption.

(c) Providing rigorous security performance validation: Through extensive simulation experiments and comprehensive security analyses (encompassing statistical property analysis, key sensitivity analysis, differential attack analysis, information entropy analysis, and correlation analysis), the proposed encryption algorithm is proven to possess high security and can effectively resist various known attacks.

The remainder of this paper is structured as follows: Section 2 meticulously describes the mathematical model of the proposed 2D-ASM chaotic system, followed by an in-depth analysis and evaluation of its dynamical characteristics. Section 3 expounds on the DNA encoding rules and associated operational definitions employed in the algorithm. Section 4 elucidates the detailed implementation steps of the image encryption algorithm integrating 2D-ASM and dynamic DNA operations, alongside the corresponding decryption process. Section 5 presents relevant simulation results, conducts comprehensive security performance analyses, and juxtaposes the proposed algorithm with other existing algorithms. Finally, Section 6 provides a conclusive summary.

## 2. The 2D-ASM Chaotic System

This section elaborates on the detailed construction of the 2D-ASM chaotic map. A series of randomness tests are conducted to evaluate its chaotic stability, chaotic characteristics, and practical usability. Furthermore, Table 1 presents seven state-of-the-art existing chaotic systems for performance comparison within this section.

### 2.1. The Ackley Function

The Ackley function, proposed by Ackley, serves as a classic multimodal test function. This function is distinguished by its expansive search domain and periodic oscillatory behavior, which together generate a complex landscape of numerous local optima. As a result, it presents substantial challenges for algorithms attempting to locate the global optimum, making it a pivotal benchmark in the study of optimization strategies. The mathematical formulation of the n-dimensional Ackley function is presented in Equation (1):

(1) $$f\left(x\right)=-20\cdot \exp\left(-0.2\sqrt{\frac{1}{n}\sum_{i=1}^{n}x_{i}^{2}}\right)-\exp\left(\frac{1}{n}\sum_{i=1}^{n}\cos\left(2\pi x_{i}\right)\right)+20+e$$

where n represents the dimension of the search space, and $x_{i}\in [-5,5]$ indicates the search range. In the two-dimensional case (d=2), the Ackley function can be expressed by Equation (2):

(2) $$f\left(x_{1},x_{2}\right)=-20\cdot e^{-0.2\sqrt{\frac{x_{1}^{2}+x_{2}^{2}}{2}}}-e^{\frac{cos\left(2\pi x_{1}\right)+cos\left(2\pi x_{2}\right)}{2}}+20+e$$

**Figure 1:**
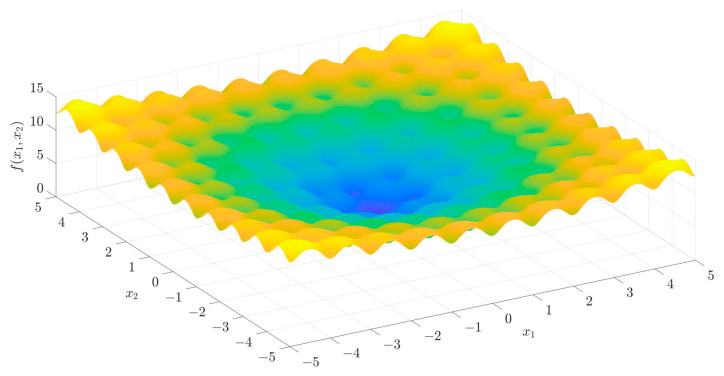
3D surface of Ackley function.

The 3D surface plot of the Ackley function is shown in Figure 1. The multimodal characteristics can be easily observed. In addition, we can observe a global optimum basin near (0,0), where the function reaches its minimum value, f(0,0)=0. In regions distant from (0,0), the function exhibits elevated values and periodic oscillatory patterns, both of which exacerbate the optimization challenge.

### 2.2. The Construction of Ackley-Sine Chaotic System

The detailed mathematical formulation of the proposed novel Ackley-Sine hyperchaotic system is presented as follows:

(3) $$\left\{\begin{array}{l} x_{i+1}=\sin\left(-20\cdot e^{a\cdot \left(-0.2\cdot \sqrt{\frac{x_{i}^{2}+y_{i}^{2}}{2}}\right)}-e^{b\cdot \left(\frac{\cos\left(2\pi x_{i}\right)+\cos\left(2\pi y_{i}\right)}{2}\right)}+20+e\right)\\ y_{i+1}=\cos\left(-20\cdot e^{b\cdot \left(-0.2\cdot \sqrt{\frac{x_{i+1}^{2}+y_{i}^{2}}{2}}\right)}-e^{a\cdot \left(\frac{\cos\left(2\pi x_{i+1}\right)+\cos\left(2\pi y_{i}\right)}{2}\right)}+20+e\right) \end{array}\right.$$

This chaotic system is derived from the above-mentioned Ackley function, integrating sine and cosine nonlinear transformations. This system integrates an exponential decay factor, which elevates the system’s nonlinearity, rendering the interaction between $x_{i+1}\;$ and $y_{i+1}$ more intricate. In contrast to traditional linear or exponential chaotic maps, this system circumvents the potential degradation of chaos induced by single exponential decay, while enhancing its nonlinear characteristics and thereby reinforcing its unpredictability. Moreover, this system features flexible control parameters a and b, allowing for dynamic tuning of its chaotic characteristics. When the control parameters $a,b\in \left(0,+\infty \right)$, the system’s phase space trajectory exhibits high chaotic features. Owing to the coupling between the exponential term and trigonometric functions, minor parameter adjustments can elicit substantial dynamical shifts, thereby rendering the system more suitable for applications including encryption and pseudo-random number generation.

When compared with traditional chaotic systems such as the Logistic map, Henon map, and Lorenz system, this system exhibits a broader chaotic regime, a wider parameter tuning range, and demonstrates enhanced complexity and randomness. The Ackley function contributes inherent multimodal characteristics and periodic oscillatory behavior (Figure 1), which introduce multiple local optima and nonlinear variations into the system. When embedded with sine/cosine trigonometric transformations, the system gains strong nonlinear coupling between state variables $\left(x_{i+1}\right)$ and $\left(y_{i+1}\right)$, avoiding the weak dynamic complexity of single-function chaotic maps (e.g., Logistic map with narrow chaotic ranges). By integrating exponential and trigonometric functions, it circumvents the short periodic windows and low-entropy domains prevalent in conventional chaotic systems. The integration of an exponential decay factor $\left(e^{-\sqrt{\frac{1}{2}\left(x_{i}^{2}+y_{i}^{2}\right)}}\right)$ further amplifies nonlinearity: it modulates the amplitude of trigonometric functions dynamically, making the interaction between state variables more intricate and preventing the “periodic window” phenomenon common in traditional chaotic maps. Compared to simpler 2D maps, the 2D-ASM’s combination of Ackley’s multimodality, trigonometric nonlinearity, and exponential modulation expands the chaotic range and enhances ergodicity, thereby achieving higher dynamic complexity. These hyperchaotic characteristics hold significant promise for applications in image encryption, secure communication, and chaos control.

### 2.3. Chaotic Performance of 2D-ASM

The dynamics of chaotic systems are assessed via a range of dynamical indicators, including bifurcation diagrams, phase portraits, Lyapunov exponents (LE), Sample Entropy (SE), Permutation Entropy (PE), Kolmogorov Entropy (KE), the 0–1 test for chaos, and Correlation Dimension (CD). Furthermore, the NIST SP 800 series of randomness tests are integrated to offer a comprehensive evaluation of stochastic properties. Collectively, these indicators serve to characterize the chaotic behavior and performance of the 2D-ASM.

#### 2.3.1. Bifurcation and Phase Diagrams

**Figure 2:**
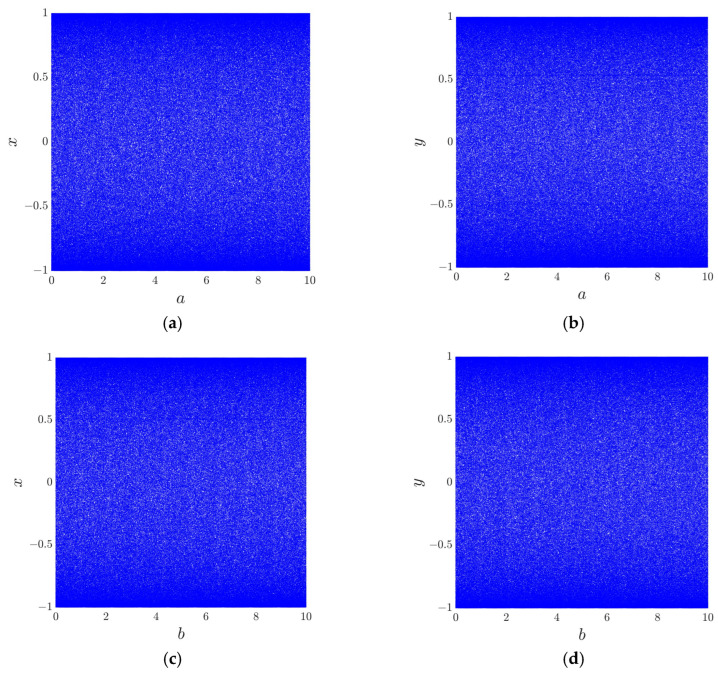
Bifurcation diagrams: (**a**) change of state variable x with parameter a; (**b**) change of state variable y with parameter a; (**c**) change of state variable x with parameter b; (**d**) change of state variable y with parameter b.

The bifurcation diagram is a tool used to illustrate the long-term behavior of a chaotic system. Figure 2 presents the two-dimensional bifurcation diagrams of the proposed 2D-ASM corresponding to its control parameters a and b. The initial position of the system is set as $\left(x_{0},y_{0}\right)=\left(0.3,\;0.6\right)$. These plots clearly show that the distributions in the bifurcation diagrams of the 2D-ASM are uniformly dispersed without any clustering phenomena across a wide range of parameters a and b. This indicates that the 2D-ASM exhibits low dependence on parameter ranges and demonstrates excellent chaotic properties.

**Figure 3:**
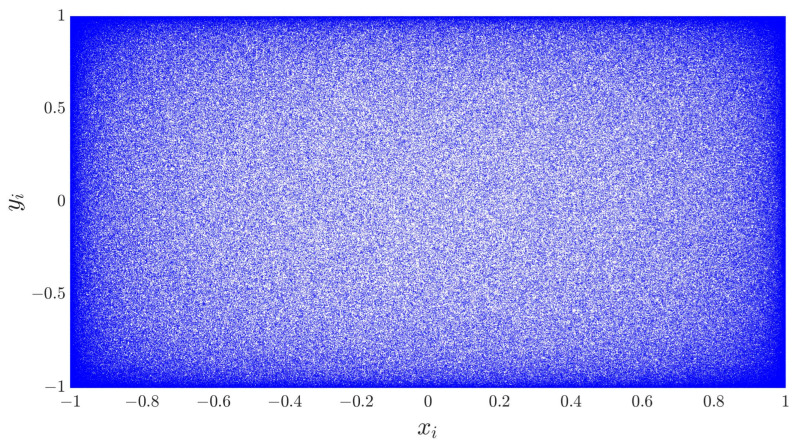
Phase diagram.

The phase trajectory diagram serves as an illustrative tool for capturing the behavior of a chaotic map through the distribution of consecutive map outputs. Figure 3 presents the phase space trajectory of the system with control parameters (a,b) set to (10, 10). While the sine and cosine functions at the heart of the 2D-ASM naturally constrain the trajectory within the range of (−1, 1), the resulting near-uniform distribution within this bounded space proves to be a desirable characteristic for our intended cryptographic application. The coupling between the exponential term and trigonometric functions ensures that the system thoroughly explores the phase space even for moderate parameter values. The Ackley function’s periodicity and the sine/cosine functions’ boundedness prevent trajectory clustering, while the exponential decay factor modulates the trajectory density to maintain uniformity—this design inherently avoids ergodicity degradation. This near-uniformity ensures that the chaotic system thoroughly explores its entire accessible phase space, promoting a highly sensitive dependence on initial conditions and parameters. This, in turn, enhances the diffusion properties of our encryption scheme, ensuring that any small change in the plaintext will rapidly propagate throughout the entire ciphertext. The uniform and dense coverage of the space also guarantees a more even distribution of key values. This inherent ergodicity, coupled with its deterministic nature, makes it well-suited for cryptographic applications.

#### 2.3.2. Lyapunov Exponent

The most effective metric for evaluating chaotic performance is the Lyapunov exponent (LE), which quantifies the sensitivity of a map to small perturbations in its initial conditions [48]. In essence, the LE describes the rate at which adjacent trajectories diverge over time in a nonlinear dynamical system. Specifically, a positive LE indicates that the system is chaotic: trajectories of a system with a positive LE will diverge rapidly even if their initial states are very close. The larger the value, the faster the orbital divergence and the higher the degree of chaos exhibited by the system. If a system has two or more positive LEs, it is classified as a hyperchaotic system. In such cases, trajectories in the phase space diverge from each other more rapidly, and the system exhibits more complex dynamical behavior. Conversely, a system with no positive LEs is in a periodic state. The detailed computational process for the Lyapunov exponent is described in ref. [49].

For a two-dimensional chaotic system, it can typically be expressed as

(4) $$\tau (x,y)=\left\{\begin{array}{c} x_{i+1}=f_{1}(x_{i},y_{i})\\ y_{i+1}=f_{2}(x_{i},y_{i}) \end{array}\right.$$

First, the Jacobian matrix J of the above map can be calculated using the partial derivatives of the mapping functions with respect to different variables:

(5) $$J(x_{i},y_{i})=\left[\begin{array}{cc} \frac{\partial f_{1}(x_{i},y_{i})}{\partial x_{i}} & \frac{\partial f_{1}(x_{i},y_{i})}{\partial y_{i}}\\ \frac{\partial f_{2}(x_{i},y_{i})}{\partial x_{i}} & \frac{\partial f_{2}(x_{i},y_{i})}{\partial y_{i}} \end{array}\right]$$

Second, the eigenvalues $\lambda_{1}$ and $\lambda_{2}$ of the Jacobian matrix J can be obtained. Then, the Lyapunov exponents can be calculated using Equation (6):

(6) $${LE}_{k}=\frac{1}{N}\underset{N\to \infty }{\lim}\sum_{i=1}^{N-1}\ln\left|\lambda_{i,k}(J)\right|$$

where *N* denotes the number of iterations of the chaotic system, $\lambda_{i,k}\left(J\right)$ are the eigenvalues of the Jacobian matrix J, and ∑ is the sum over all iteration steps of the logarithm of the eigenvalues. In practical computations, a large number of iterations are typically employed to approximate the limit value.

**Figure 4:**
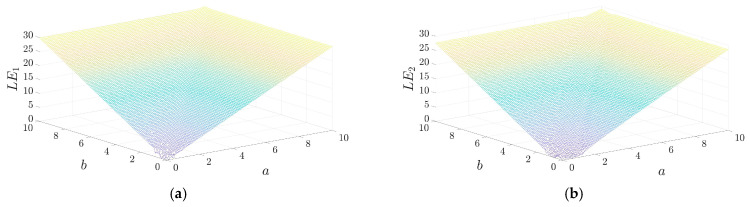
3D LEs variation of 2D-ASM according to the control parameters a, b. (**a**) LE1, (**b**) LE2.

Figure 4 shows the 3D surface plot of Lyapunov exponents (LE_1_ and LE_2_) for the 2D-ASM proposed in this paper. The horizontal and vertical axes represent control parameters a and b, respectively. It can be observed that both LEs exhibit large positive values. This indicates that the proposed 2D-ASM is a hyperchaotic system and sensitive to initial conditions. In addition, LE_1_ and LE_2_ increase as parameters a and b increase. All This makes the system very suitable for image encryption.

**Figure 5:**
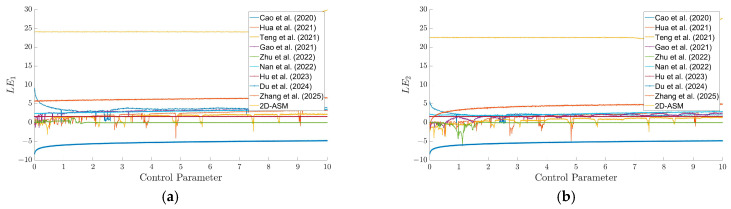
Comparison of LE according to the control parameter for existing chaotic maps. (**a**) LE1, (**b**) LE2.

To further verify the advantages of the 2D-ASM, Figure 5 presents comparative curves of the Lyapunov exponents (LE_1_ and LE_2_) for the 2D-ASM in comparison with those of other chaotic systems. The 2D-ASM exhibits the largest LE value, exceeding 23, which indicates that it demonstrates superior chaotic dynamical behavior and higher key sensitivity. Moreover, in contrast to the abrupt changes or discontinuities observed in the LE values of some comparative systems, the LEs of the 2D-ASM maintain better stability across the entire parameter space. This property enables the 2D-ASM to retain excellent chaotic properties under different parameter configurations, thereby facilitating the construction of more robust encryption systems.

#### 2.3.3. Sample Entropy

Sample Entropy (SE) is an indicator used to measure the complexity of a time series, and its value is positively correlated with the degree of chaos in the system [50]. The core concept of SE is to quantify the self-similarity of a system over time, or the predictability of the system’s dynamic behavior across different time scales. Generally, a larger Sample Entropy value indicates weaker regularity, a higher degree of chaos, and greater complexity in the system. Conversely, a lower SE value indicates that the system is more regular. For a given time series $x\;=\;x_{1},x_{2},\ldots ,x_{N}$ of length N, the calculation of Sample Entropy (SE) proceeds as follows:

Given an embedding dimension m, multiple m-dimensional subsequences can be constructed as $x\left(i\right)=\left\{x_{i},x_{i+1},\ldots ,x_{i+m-1}\right\},\;i=1,2,\ldots ,N-m+1$. These subsequences are used to quantify the similarity of the time series across different time windows. For any two subsequences x(i) and x(j), calculate the Chebyshev distance between them, which measures the maximum deviation of data points within the two-time windows. The detailed formula is presented in Equation (7):

(7) $$d\left(x\left(i\right),x\left(j\right)\right)=\underset{k=0,\ldots ,m-1}{\max}\left|x_{i+k}-x_{j+k}\right|$$

Set a tolerance threshold r: if the Chebyshev distance between two subsequences is less than r, they are deemed similar. The number of subsequence pair $C_{i}^{m}\left(r\right)$ satisfying this condition is counted, as defined in Equation (8):

(8) $$A_{i}^{m}\left(r\right)=\frac{1}{N-m+1}\sum_{j=1}^{N-m+1}\left[\theta \left(r-d\left(x\left(i\right),x\left(j\right)\right)\right)\right]$$

Then, the average similarity ratios under dimensions m and m + 1 are calculated by Equation (9) and Equation (10), respectively:

(9) $$A^{m}\left(r\right)=\frac{1}{N-m+1}{\sum_{i=1}^{N-m+1}A}_{i}^{m}\left(r\right)$$

(10) $$A^{m+1}\left(r\right)=\frac{1}{N-m}\sum_{i=1}^{N-m}A_{i}^{m+1}\left(r\right)$$

Finally, the Sample Entropy can be defined as

(11) $$SE=-\ln\left(\frac{A^{m+1}\left(r\right)}{A^{m}\left(r\right)}\right)$$

The intuitive meaning of Sample Entropy is the negative logarithm of the rate of change in the number of similar subsequences in the time series when the dimension increases from m to m+1. A larger SE value indicates that the system has lower predictability and higher complexity.

**Figure 6:**
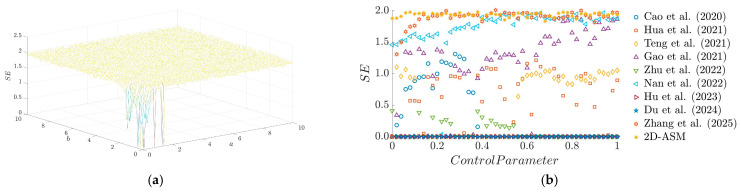
SE results. (**a**) 3D plot of SE, (**b**) Comparison of LE for existing chaotic maps.

The SE results of the 2D-ASM as a function of control parameters are presented in Figure 6. It can be observed that the system exhibits large SE values across an extensive parameter space, indicating favorable complexity and randomness—this lays a solid foundation for subsequent image encryption applications. Furthermore, a comparison of SE values between the 2D-ASM and other chaotic systems reveals that the 2D-ASM holds significant advantages in maintaining high SE values. This underscores its superiority in generating more complex keystreams and enhancing resistance to attacks.

#### 2.3.4. Permutation Entropy

Permutation Entropy (PE) is a fundamental complexity measure for time series, proposed by Bandt and Pompe in 2002 [51]. It captures hidden nonlinear characteristics by analyzing the permutation patterns of values within the time series. In general, a higher PE value indicates that the sequence is closer to a random state and exhibits greater complexity. For a given time series $x\;=\;x_{1},x_{2},\ldots ,x_{N}$ of length N, the calculation steps for PE are as follows:

Select an appropriate embedding dimension m and time delay τ, then construct multiple subsequences of length m from the original time series as follows: $x\left(i\right)=x_{i},x_{i+\tau },x_{i+2\tau },\ldots ,x_{i+\left(m-1\right)\tau },\;i=1,2,\ldots ,N-\left(m-1\right)\tau$. Here, τ governs the time interval between data points in the subsequence, while m affects the complexity of sequence patterns. For each subsequence, sort its elements in ascending order by their values and record the relative positions (indices) of the data points. For instance, if x(i) = {3.1, 1.2, 5.6}, sorting yields {1.2, 3.1, 5.6}, with the corresponding permutation pattern being (2, 1, 3)—this is because the second element is the smallest, the first is intermediate, and the third is the largest. Count the occurrences of all possible permutation patterns across the entire time series, then normalize these counts to obtain a probability distribution. The calculation formula for PE is given in Equation (12):

(12) $$PE=-\frac{1}{\log_{2}m!}\sum_{i=1}^{m!}p_{i}\left(\pi \right)\log_{2}p_{i}\left(\pi \right)$$

where m denotes the embedding dimension, which represents the length of the segmented time series segments. $p_{i}\left(\pi \right)$ stands for the occurrence probability of the permutation pattern π in the time series. The ${log}_{2}m!$ term serves as a normalization factor, ensuring that the Permutation Entropy value lies within the range [0, 1], and thus facilitating comparisons across different scales.

**Figure 7:**
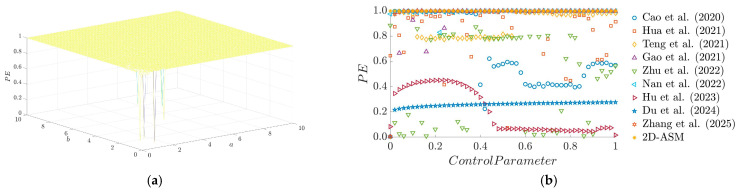
PE results. (**a**) 3D plot of PE, (**b**) Comparison of PE for existing chaotic maps.

Figure 7a illustrates the distribution of PE for the 2D-ASM system under varying control parameters a and b. As observed from the figure, the PE value remains close to 1 across most parameter regions, indicating that the system exhibits strongly chaotic characteristics under these conditions. Meanwhile, Figure 7b presents a comparison of PE between the 2D-ASM system and several existing systems. It can be seen that the 2D-ASM maintains consistently high PE values (close to 1) throughout the entire range of control parameters, demonstrating stronger chaotic robustness as well as its capacity to sustain high complexity and unpredictability under diverse conditions.

#### 2.3.5. Kolmogorov Entropy

Kolmogorov Entropy (KE) is another well-established metric for quantifying the complexity or randomness of a dynamical system [52]. It characterizes the rate at which information is lost during the system’s evolution, with its magnitude directly reflecting the predictability of the system’s future behavior. If the KE value is 0, the system is insensitive to small perturbations in initial conditions, accompanied by minimal information loss—this indicates relative predictability. When the KE value exceeds 0, the system becomes sensitive to variations in initial information: its trajectories diverge exponentially over time, thereby exhibiting chaotic characteristics. A larger KE value corresponds to higher system complexity and lower predictability of future states. The calculation of KE is grounded in the growth rate of Shannon entropy, with its mathematical expression provided in Equation (13):

(13) $$KE=\underset{l\to \infty }{\lim}\frac{H_{l}}{l},H_{l}=-\sum P\left(i_{1},i_{2},\ldots ,i_{l}\right)\ln P\left(i_{1},i_{2},\ldots ,i_{l}\right)$$

where l represents the embedding dimension, and $P\left(i_{1},i_{2},\ldots ,i_{l}\right)$ is the probability distribution of the system state sequence.

**Figure 8:**
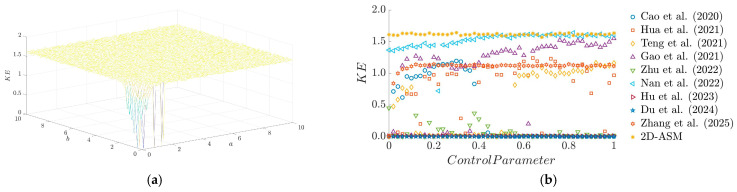
KE results. (**a**) 3D plot of PE, (**b**) Comparison of KE for existing chaotic maps.

Figure 8a illustrates the distribution of KE for the 2D-ASM system as control parameters a and b vary. As evident from the 3D surface plot, the 2D-ASM exhibits high KE values across most of the parameter space, indicating that the system possesses considerable complexity and randomness under most parameter combinations. Furthermore, as shown in Figure 8b, the KE curve of the 2D-ASM is significantly higher than those of other chaotic systems over most parameter ranges. This suggests that the 2D-ASM can generate sequences with higher complexity, thereby enhancing the security of the encryption algorithm.

#### 2.3.6. 0–1 Test

The 0–1 test is a method that directly determines whether the underlying dynamic system behind a time series is regular or chaotic based on the time series itself [53]. Originally proposed by Gottwald and Melbourne in 2004, it was further refined in 2009. This method operates directly on time series data and does not require phase space reconstruction, thus avoiding the difficulty of selecting parameters such as embedding dimension and time delay. The method is computationally efficient in practical applications and is well suited for analyzing time series of limited length. The result of the 0–1 test should be approximately equal to 1 for a chaotic system or approximately equal to 0 for a non-chaotic system.

The basic idea is that a time series $\varphi \left(j\right)$ drives a two-dimensional translational dynamical system, whose equations are shown below (Equation (14)):

(14) $$\begin{array}{l} p\left(n\right)=\sum \left[\phi \left(j\right)\times \cos\left(\theta \left(j\right)\right)\right]\\ q\left(n\right)=\sum \left[\phi \left(j\right)\times \sin\left(\theta \left(j\right)\right)\right] \end{array}$$

where $\theta \left(j\right)=j\times c$, and c is a fixed irrational number. Then, the mean square displacement $M\left(n\right)$ is calculated. The asymptotic behavior of $M\left(n\right)$ can reveal dynamics. To obtain a clear output, the growth rate K is further calculated, with its computational formula as shown in the following equation (Equations (15) and (16)):

(15) $$M\left(n\right)=\underset{n\to \infty }{\lim}\frac{1}{N}\sum \left[{\left(p\left(j+n\right)-p\left(j\right)\right)}^{2}+{\left(q\left(j+n\right)-q\left(j\right)\right)}^{2}\right]$$

(16) $$K=\underset{n\to \infty }{\lim}\frac{\log M\left(n\right)}{\log\left(n\right)}$$

If the value of K is close to 0: This indicates that the displacement is bounded, and the $\varphi \left(j\right)$ comes from a regular (periodic or quasi-periodic) system. If the value of K is close to 1: This indicates that the displacement is unbounded, similar to Brownian motion, and the $\varphi \left(j\right)$ comes from a chaotic system.

**Figure 9:**
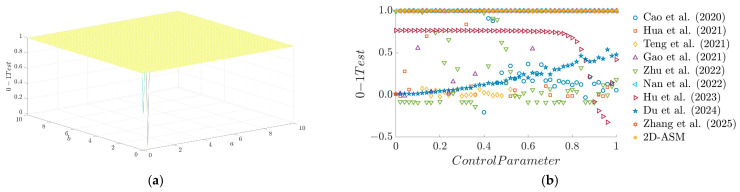
0–1 test result. (**a**) 3D plot of 0–1 test, (**b**) comparison of 0–1 test for existing chaotic maps.

The 0–1 test results presented in Figure 9a indicate that the 2D-ASM yields values closer to the ideal value of 1 across a wide parameter range. As further observed in Figure 9b, when compared to other chaotic systems, it exhibits more superior and consistently stable chaotic properties.

#### 2.3.7. Correlation Dimension

The correlation dimension (CD) is a key fractal characteristic that describes the geometric complexity of a dynamic system’s attractor [54]. It quantifies the distribution of points in phase space, thereby reflecting the system’s chaotic properties and complexity. A higher CD value indicates a more complex and unpredictable system. The CD is primarily calculated using the correlation integral method, which characterizes the correlation degree between points on the system’s trajectory across different scales R. The correlation integral C(R), representing the geometric correlation between pairs of points in the system, is defined in Equation (17):

(17) $$C\left(R\right)=\frac{2}{N\left(N-1\right)}\sum_{i=1}^{N}N_{i}\left(R\right)$$

where N is the total number of points in the phase space, and $N_{i}\left(R\right)$ represents the number of neighboring points within a radius R of point i. The calculation of $N_{i}\left(R\right)$ is given in Equation (18):

(18) $$N_{i}\left(R\right)=\sum_{k=1,k\neq i}^{N}1\left(\left\|Y_{i}-Y_{k}\right\|<R\right)$$

This formula can be interpreted as follows: for the i-th point, it calculates the number of all other points within a distance R, where $N_{r}$ denotes the number of scales. To select appropriate values for R, a logarithmic spacing method is typically employed, as shown in Equation (19):

(19) $$R=\exp\left(linspace\left(\log\left(r_{\min}\right),\log\left(r_{\max}\right),N_{r}\right)\right)$$

In the equation, $r_{min}$ and $r_{\;max}$ denote the minimum and maximum scales, respectively. Using a logarithmic scale ensures that a sufficient range is covered during the calculation. For the practical calculation of CD, linear regression fitting method—as shown in Equation (20)—is employed to estimate $D_{2}$ from the slope in logarithmic coordinates:

(20) $$D_{2}\approx \frac{d\log C\left(R\right)}{d\log R}$$

**Figure 10:**
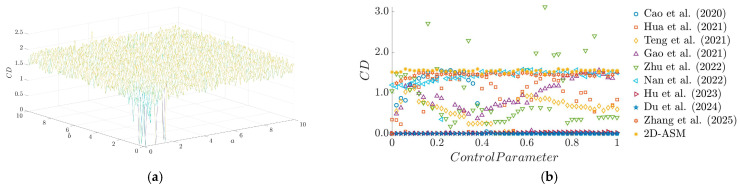
CD results. (**a**) 3D plot of PE, (**b**) comparison of CD for existing chaotic maps.

Figure 10a shows that the 2D-ASM exhibits relatively high CD values across most parameter regions, with the overall surface displaying significant fluctuations. This indicates that the CD value is highly sensitive to parameter changes, a characteristic advantageous for chaotic encryption systems. As shown in the analysis results of Figure 10b, within the examined parameter range, the 2D-ASM demonstrates a significantly higher correlation dimension than other systems, indicating that its chaotic dynamics possess greater complexity and more effective information diffusion capabilities.

#### 2.3.8. NIST 800

The NIST test suite is a set of statistical tests developed by the U.S. National Institute of Standards and Technology (NIST), specifically designed to evaluate the randomness and performance of random number generators (RNGs) [55]. This suite encompasses various statistical methods, including the frequency test, runs test, and autocorrelation test, among others. By analyzing diverse statistical properties of a random sequence, it assesses whether the sequence meets the required standards for randomness. In the field of cryptography, NIST tests are widely employed to examine the security of pseudo-random sequences generated by encryption algorithms, ensuring their resistance to attacks and effectiveness in information hiding. Furthermore, they serve as an important tool for evaluating the randomness of chaotic systems, used to measure the complexity and unpredictability of sequences generated thereby. The applications of NIST tests extend beyond cryptography and information security, playing significant roles in areas such as random number simulation, numerical computation, and physical experiments.

This paper employs NIST statistical test methods to verify the randomness of sequences generated by the 2D-ASM system. In the tests, the sequence length is set to 1,000,000, the initial values $\left(x_{0},y_{0}\right)$ are set to (0.3, 0.6), the control parameters a and b are uniformly set to 10, and the significance level α is set to 0.01. Table 2 summarizes the test results, showing that the 2D-ASM sequence successfully passed all 15 NIST tests, preliminarily confirming that it possesses good randomness. Compared to other chaotic sequences, the 2D-ASM sequence exhibited higher stability in the NIST tests, underscoring its potential for application in random sequence generation.

In summary, leveraging its superior chaotic characteristics and complex dynamical behavior, the trajectory of the 2D-ASM system exhibits extremely high randomness and unpredictability. Furthermore, through analysis and comparison using multi-dimensional metrics—including Lyapunov exponents (LE), Sample Entropy (SE), Permutation Entropy (PE), Kolmogorov Entropy (KE), correlation dimension (CD), the 0–1 test, and randomness tests—it is verified that, compared to existing two-dimensional chaotic systems, the 2D-ASM not only covers a wider chaotic range but also demonstrates superior random ergodicity and stronger unpredictability. Overall, these analysis results indicate that 2D-ASM possesses good chaotic properties, making it suitable for constructing high-security encryption systems.

## 3. DNA Coding and Operations

DNA coding and operational rules are primarily used in information storage and cryptography, utilizing the four bases in DNA—A (Adenine), T (Thymine), C (Cytosine), and G (Guanine)—for data representation and computation. Typically, a binary mapping method is employed where 00, 01, 10, and 11 correspond to A, C, G, and T, respectively, thereby enabling data encoding, as detailed in Table 3. In DNA operations, complementary pairing is a fundamental rule: A pairs with T, and C pairs with G. This property can be utilized for encryption and data recovery. Additionally, DNA computation encompasses operations such as XOR, addition, and subtraction. The DNA computations used in this algorithm are presented in Table 4, Table 5 and Table 6, which achieve encryption through base correspondence rules, rendering the data more resistant to cracking. Furthermore, DNA coding has extensive applications in information security, including key expansion, image encryption, steganography, and data storage. Among these, DNA encryption typically employs a method that converts plaintext into a DNA sequence and then uses DNA logic operations for encryption, providing keys with higher randomness and stronger attack resistance. Meanwhile, DNA possesses ultra-high storage density and high security, making it a promising future direction for encryption technology.

## 4. Proposed ASM-IE Scheme

**Figure 11:**
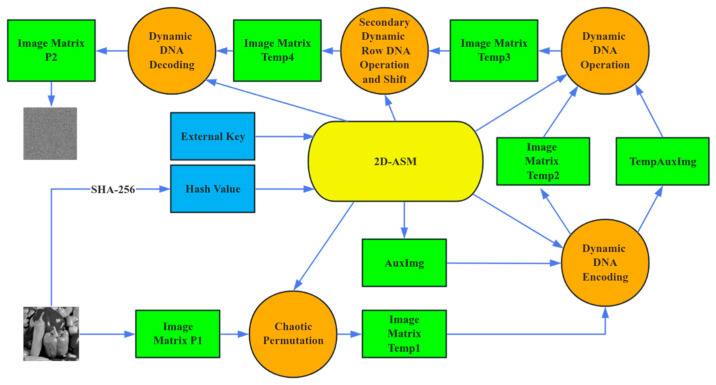
Encryption flowchart.

In the image encryption algorithm, the SHA-256 hash value of the original image P together with four external keys ($\mu_{i}$) is first utilized to initialize the 2D-ASM chaotic system. Then, six chaotic sequences ($S_{x1}$, $S_{x2}$, $S_{x3}$, $S_{y1}$, $S_{y2}$, $S_{y3}$) are generated through iteration, which are subsequently transformed into $S_{x1}^{\prime }$, $S_{x2}^{\prime }$, $S_{x3}^{\prime }$, $S_{y1}^{\prime }$, and $S_{y2}^{\prime }$, $S_{y3}^{\prime }$ according to Equations (20)–(24). Next, pixel permutation is performed on image P using $S_{x1}^{\prime }$ to obtain temp1. The process then enters a multi-layer diffusion stage: auxiliary data AuxImg is generated using $S_{x2}^{\prime }$; with dynamically selected rules based on $S_{x3}^{\prime }$ and $S_{y1}^{\prime }$, temp1 and AuxImg are DNA-encoded into temp2 and TempAuxImg; $S_{y2}^{\prime }$ controls the DNA-level operations (such as addition, XOR, etc.) between temp2 and TempAuxImg to produce temp3; afterward, $S_{y3}^{\prime }$ is used to perform a secondary, row-by-row dynamic DNA operation on temp3. Based on the value of $S_{y3}^{\prime }$ (0–3), the specific type of DNA base operation within intra-row units is determined, after which the corresponding row transformation (circular right shift, circular left shift, base swapping, or no operation) is applied, resulting in temp4. Finally, temp4 is decoded using $S_{x3}^{\prime }$ to obtain the final encrypted image, crypt. The decryption process is the inverse of the steps described above. The specific encryption flowchart is presented in Figure 11.

### 4.1. Encryption Procedure

Step 1: Construct Hash Value

Calculate the 256-bit hash digest $H=\{h_{1},h_{2},\ldots ,h_{32}\}$ of the original image P (m × n) using the SHA-256 algorithm. (Each $h_{i}$ is 8 bits).

Step 2: Generate Parameters and Initial Values for Chaotic Sequence

The control parameters a and b, and the initial states $x_{0}$ and $y_{0}$ of the 2D-ASM chaotic system (see Equation (3)) are jointly determined by the image hash value and four externally set keys ($\mu_{1},\mu_{2},\mu_{3},\mu_{4}$), as shown in the following Equation (21):

(21) $$\left\{\begin{array}{l} x_{0}=\frac{1}{255}\left(\left(\sum_{i=1}^{8^{⊕}}h_{i}\right)⊕\mu_{1}\right),y_{0}=\frac{1}{511}\left(\left(\sum_{i=9}^{16^{⊕}}h_{i}\right)⊕\mu_{2}\right)\\ a=\frac{1}{15}\left(\left(\sum_{i=17}^{24^{⊕}}h_{i}\right)⊕\mu_{3}\right),b=\frac{1}{15}\left(\left(\sum_{i=25}^{32^{⊕}}h_{i}\right)⊕\mu_{4}\right) \end{array}\right.$$

Step 3: Generate Chaotic Sequences

Substitute the parameters a and b and the initial states x_0_ and y_0_ obtained from Equation (21) into the 2D-ASM system and iterate $N_{1}\;+\;3MN$ times ($N_{1}\;$ = 1,000,000) to generate two chaotic sequences, $S_{x}$ and $S_{y}$, each of length $N_{1}\;+\;3MN$. Discard the first $N_{1}$ points of $S_{x}$ and $S_{y}$ to eliminate transient effects. Then, derive six chaotic sequences, $S_{x1}$, $S_{x2}$, $S_{x3}$, $S_{y1}$, $S_{y2}$, and $S_{y3}$, each of length MN, from the remaining parts of $S_{x}$ and $S_{y}$, respectively.

Step 4: Pixel Permutation

Process the chaotic sequence $S_{x1}$ using Equation (22) to map its original chaotic values into the integer range [1, m×n], obtaining $S_{x1}^{\prime }$. Apply an index selection and swapping mechanism based on the processed $S_{x1}^{\prime }$ to obtain the permuted image temp1 (m×n).

(22) $$S_{x1}^{\prime }\left(i\right)=\left(\left\lfloor \left|S_{x1}\left(i\right)\cdot 10^{10}\right|\right\rfloor \mathrm{mod}\left(m\cdot n\right)\right)+1$$

Step 5: Auxiliary Data Generation and Dynamic DNA Encoding

Auxiliary data AuxImg, which plays a critical role in strengthening the diffusion process, is generated in this step. Unlike a random image matrix, AuxImg is derived from the chaotic sequence $S_{x2}$, adjusted according to Equation (23), ensuring it is not entirely random but rather dependent on the chaotic system and key. The generation process uses dynamically selected rules based on $S_{x3}^{\prime }$ and $S_{y1}^{\prime }$ (where $S_{x3}^{\prime }$ and $S_{y1}^{\prime }$ are derived from $S_{x3}$ and $S_{y1}^{\prime }$ according to Equation (22)), as detailed in Equation (24) and Table 3, further introducing nonlinearity and complexity. By encoding both the permuted image temp1 and AuxImg using specific DNA encoding rules (Table 3), the algorithm ‘mixes’ the data from the original image with AuxImg, resulting in DNA sequences temp2 and TempAuxImg. This mixing of data, driven by the chaotic system and dynamic rules, enhances the diffusion effect and makes the algorithm more resistant to cryptanalysis.

(23) $$\left\{\begin{array}{l} S_{x2}^{\prime }\left(i\right)=\left(\left\lfloor \left|S_{x2}\left(i\right)\cdot 10^{10}\right|\right\rfloor \mathrm{mod}\left(256\right)\right)\\ AuxImg=reshape\left(S_{x2}^{\prime },m\cdot n,1\right) \end{array}\right.$$

(24) $$\left\{\begin{array}{l} S_{x3}^{\prime }\left(i\right)=\left(\left\lfloor \left|S_{x3}\left(i\right)\cdot 10^{10}\right|\right\rfloor \mathrm{mod}8\right)+1\\ S_{y1}^{\prime }\left(i\right)=\left(\left\lfloor \left|S_{y1}\left(i\right)\cdot 10^{10}\right|\right\rfloor \mathrm{mod}8\right)+1 \end{array}\right.$$

Step 6: Primary Dynamic DNA Operation

Transform the chaotic sequence $S_{y2}$ into a control subsequence $S_{y2}^{\prime }$ using Equation (25) (where the chaotic values $S_{y2}^{\prime }\left(i\right)$ are scaled to 0, 1, or 2, representing ADD, SUB, and XOR operations, respectively). Perform DNA operations on temp2 and TempAuxImg based on predefined DNA operation rules (as shown in Table 4, Table 5 and Table 6) and the control subsequence $S_{y2}^{\prime }$, obtaining the intermediate result temp3.

(25) $$S_{y2}^{\prime }\left(i\right)=\mathrm{mod}\left(\left\lfloor \left|S_{y2}\left(i\right)\cdot 10^{10}\right|\right\rfloor ,3\right)$$

Step 7: Secondary Intra-row Dynamic DNA Operation and Shift

For each row of temp3, first calculate the control subsequence $S_{y3}^{\prime }$ based on the chaotic sequence $S_{y3}$ and Equation (26) (where the chaotic values $S_{y3}^{\prime }\left(i\right)$ are scaled to 0, 1, 2, or 3). Subsequently, perform a specific operation on the row according to $S_{y3}^{\prime }\left(i\right)$:

If $S_{y3}^{\prime }\left(i\right)$ is 0: First, perform DNA operations within each 4-base unit (SUB operation between the 1st and 3rd bases, XOR operation between the 2nd and 4th bases), then perform a circular right shift on the entire row.

If $S_{y3}^{\prime }\left(i\right)$ is 1: First, perform DNA operations within each unit (ADD operation between the 1st and 4th bases, XOR operation between the 2nd and 3rd bases), then perform a circular left shift on the entire row.

If $S_{y3}^{\prime }\left(i\right)$ is 2: First, perform DNA operations within each unit (ADD operation between the 1st and 2nd bases, SUB operation between the 3rd and 4th bases), then swap the 1st and 4th bases within each unit.

If $S_{y3}^{\prime }\left(i\right)$ is 3: No DNA operation or subsequent shift/swap operation is performed on this row.

**Figure 12:**
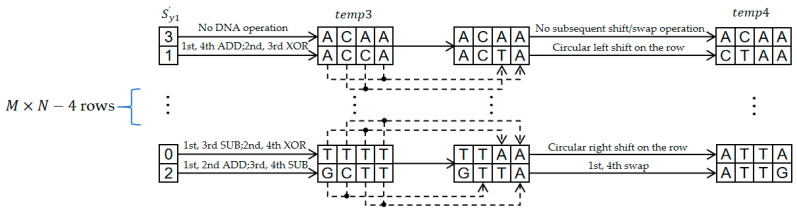
Secondary Cascade Diffusion Flowchart.

After processing all rows, temp4 is obtained, as shown in Figure 12.

(26) $$S_{y3}^{\prime }\left(i\right)=\mathrm{mod}\left(\left\lfloor \left|S_{y3}\left(i\right)\cdot 10^{10}\right|\right\rfloor ,4\right)$$

Step 8: Decoding

Decode temp4 using the chaotic control sequence $S_{x3}^{\prime }$ to obtain the final encrypted image, crypt.

### 4.2. Decryption Procedure

Following cryptographic reversibility principles, decryption is the reverse of encryption.

First, the 2D-ASM chaotic system is initialized using the same SHA-256 hash value of the original image and four external keys, generating identical chaotic sequences.

Next, the encrypted image is DNA-encoded using $S_{x3}^{\prime }$ to recover temp4. The row-wise transformations controlled by $S_{y3}^{\prime }$ are then inversely applied (circular shifts are reversed, and row swapping is performed again). Subsequently, inverse DNA operations corresponding to those used in encryption are executed to obtain temp2.

After regenerating the auxiliary image AuxImg using $S_{x2}^{\prime }$, inverse DNA diffusion operations controlled by $S_{y2}^{\prime }$ are performed to recover temp1. Finally, the inverse permutation based on $S_{x1}^{\prime }$ restores the original plaintext image.

## 5. Security Analysis of ASM-IE

This section presents a systematic simulation and analysis of the proposed encryption scheme based on the 2D-ASM map. We not only conduct a comprehensive evaluation of its security and robustness but also compare its performance with that of several recent high-performing encryption mechanisms.

### 5.1. Histogram

An image histogram characterizes the distribution pattern of pixel grayscale values in an image. Original images typically exhibit distinct grayscale distribution features, whereas an encrypted image should display a histogram close to uniform distribution to conceal the statistical characteristics of the original image. If the histogram of the encrypted image still retains obvious distribution patterns, attackers may exploit statistical analysis to recover the original image. Thus, histogram uniformity serves as a critical visual security metric for evaluating encryption effectiveness.

**Figure 13:**
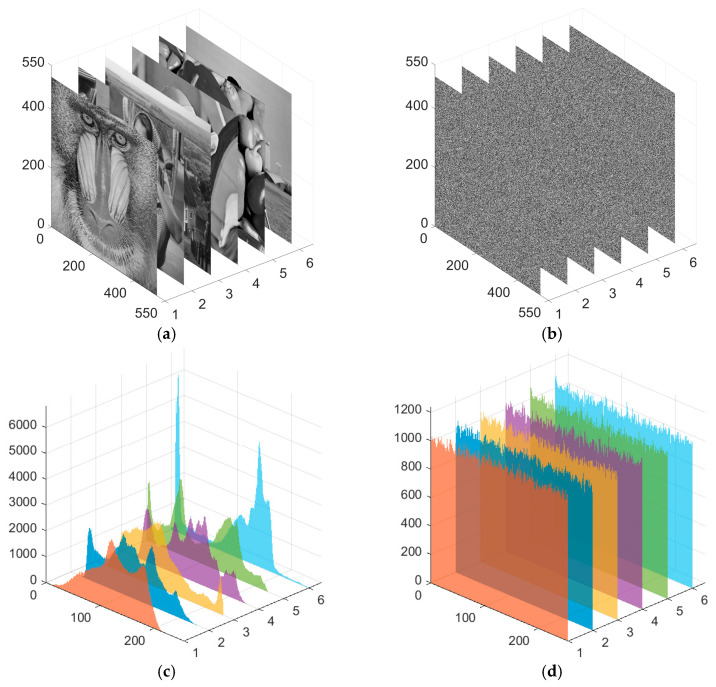
Encryption performance testing of the proposed scheme. (**a**) Origin pictures, (**b**) encrypted pictures, (**c**,**d**) histograms of origin and encrypted pictures (The image index is as follows: 1–Baboon, 2–Barbara, 3–Goldhill, 4–Lena, 5–Peppers, 6–Cameraman).

To intuitively assess the performance of the ASM-IE encryption algorithm, Figure 13 employs 3D stacked visualization to compare and display the encryption effects of six standard 512 × 512 test images before and after encryption. This set of figures clearly illustrates the algorithm’s performance across two key security dimensions: First, in terms of visual content obfuscation, the clear structures and recognizable information in original images (Figure 13a) are completely transformed into unintelligible, noise-like patterns after ASM-IE encryption (Figure 13b), effectively hiding the visual features of the plaintext. Second, regarding the alteration of statistical properties, the non-uniform grayscale histograms of original images (Figure 13c)—which typically exhibit distinct peaks, valleys, and statistical redundancy—are converted into highly flat and uniform histograms after encryption (Figure 13d). Pixel values are approximately equiprobable across the entire grayscale range, significantly enhancing resistance against statistical analysis attacks. Overall, through intuitive comparisons at both visual and statistical levels, Figure 13 strongly demonstrates that the ASM-IE algorithm can simultaneously achieve thorough obfuscation of visual information and effective randomization of the image’s statistical distribution, meet the expected security goals of image encryption, and exhibit excellent encryption performance.

### 5.2. Adjacent Pixel Correlation Analysis

In natural images, grayscale values of adjacent pixels exhibit high correlation, particularly in flat regions. In contrast, an ideal encrypted image should disrupt this correlation, reducing it to near zero. Correlation is quantified using the correlation coefficient, which ranges from [−1, 1], with a value of 0 indicating no correlation. Correlation coefficients are typically computed separately for the horizontal, vertical, and diagonal directions. The corresponding formula is provided in Equation (27):

(27) $$r=\frac{E\left[\left(x-E\left(x\right)\right)\left(y-E\left(y\right)\right)\right]}{\sqrt{D\left(x\right)}\sqrt{D\left(y\right)}}$$

where x and y represent the grayscale values of adjacent pixel pairs, E(x) is the expectation (mean), and D(x) is the variance. For an encrypted image, the correlation coefficient r should approach 0.

**Figure 14:**
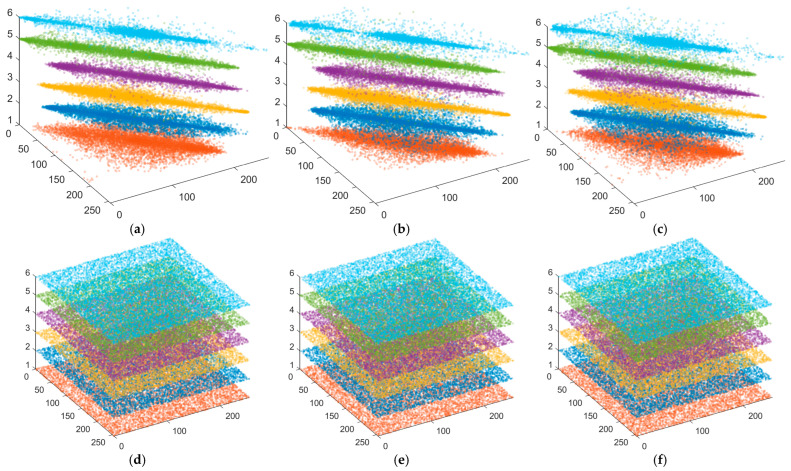
Adjacent pixel correlation distributions in the horizontal, vertical, and diagonal directions for: (**a**–**c**) the original plain image; (**d**–**f**) the corresponding encrypted ciphertext image (The image index is as follows: 1–Baboon, 2–Barbara, 3–Goldhill, 4–Lena, 5–Peppers, 6–Cameraman).

Figure 14 visually compares pixel correlation before and after encryption using 3D scatter plots: original images (Figure 14a–c) exhibit strong linear aggregation (high correlation), while points in the encrypted images (Figure 14d–f) are uniformly scattered, indicating significant elimination of correlation. The quantitative results of correlation coefficients in Table 7 further confirm this: coefficients for original images are close to 1, whereas those after encryption by the proposed algorithm drop sharply to near 0. Compared with the results of Gao [8] and Lai et al. [18] (see Table 7), the proposed method demonstrates competitive performance in reducing ciphertext correlation, with coefficient values consistently close to zero. In summary, both visual and quantitative assessments indicate that the proposed algorithm can effectively disrupt pixel correlation and resist correlation attacks.

### 5.3. Differential Attack Analysis

The differential attack is a method that attempts to recover the original image or key by analyzing the response of the ciphertext to slight changes in the plaintext. A secure image encryption algorithm should be highly sensitive to minor changes in the plaintext, causing drastic changes in the ciphertext. Two common evaluation metrics are NPCR and UACI:

NPCR (Number of Pixels Change Rate) measures how many pixels change in the encrypted image; a value closer to the ideal 100% is better. Its calculation method is shown in Equation (28):

(28) $$NPCR=\frac{\sum_{i,j}D\left(i,j\right)}{M\times N}\times 100\%,D\left(i,j\right)=\left\{\begin{array}{l} 1,if\;C_{1}\left(i,j\right)\neq C_{2}\left(i,j\right)\\ 0,otherwise \end{array}\right.$$

UACI (Unified Average Changing Intensity) quantifies the average intensity of pixel value changes; a value closer to the ideal 33.3% is preferable. Its mathematical formula is provided in Equation (29):

(29) $$UACI=\frac{1}{M\times N}\sum_{i,j}\frac{\left|C_{1}\left(i,j\right)-C_{2}\left(i,j\right)\right|}{255}\times 100\%$$

where $C_{1}$ and $C_{2}$ denote the two ciphertext images corresponding to the original plaintext and the slightly modified plaintext, respectively.

As shown in Table 8, NPCR and UACI tests were conducted on several standard test images. The results indicate that for all test images, the NPCR and UACI values are consistently close to the theoretical expected values. These numerical results strongly demonstrate that the proposed algorithm exhibits an excellent avalanche effect: even a minor change to a single pixel bit in the original image leads to ciphertext pixel changes approaching the theoretical maximum (high NPCR) and significant average intensity differences (high UACI). Overall, the proposed encryption method performs excellently in resisting differential attacks. Its NPCR and UACI values are also competitive compared to those reported by Zhu et al. [12] and Lai et al. [18]. This further confirms that the algorithm possesses strong nonlinearity and diffusion properties, enabling effective resistance against differential analysis attacks, while maintaining good stability and robustness when processing images with diverse statistical features.

### 5.4. Key Space

The security of the proposed encryption scheme is ensured by an external key set consisting of four independent parameters $(\mu_{1},\mu_{2},\mu_{3},\mu_{4}$). In both theoretical design and practical implementation, each parameter is represented as a 64-bit double-precision floating-point number. Therefore, each external key provides $2^{64}$ possible values. Since the four keys are mutually independent, the total key space of the proposed scheme is ${{(2}^{64})}^{4}=2^{256}$. Such a large key space is far beyond the commonly accepted security threshold of $2^{128}$, which is sufficient to effectively resist brute-force attacks.

### 5.5. Key Sensitivity Analysis

**Figure 15:**
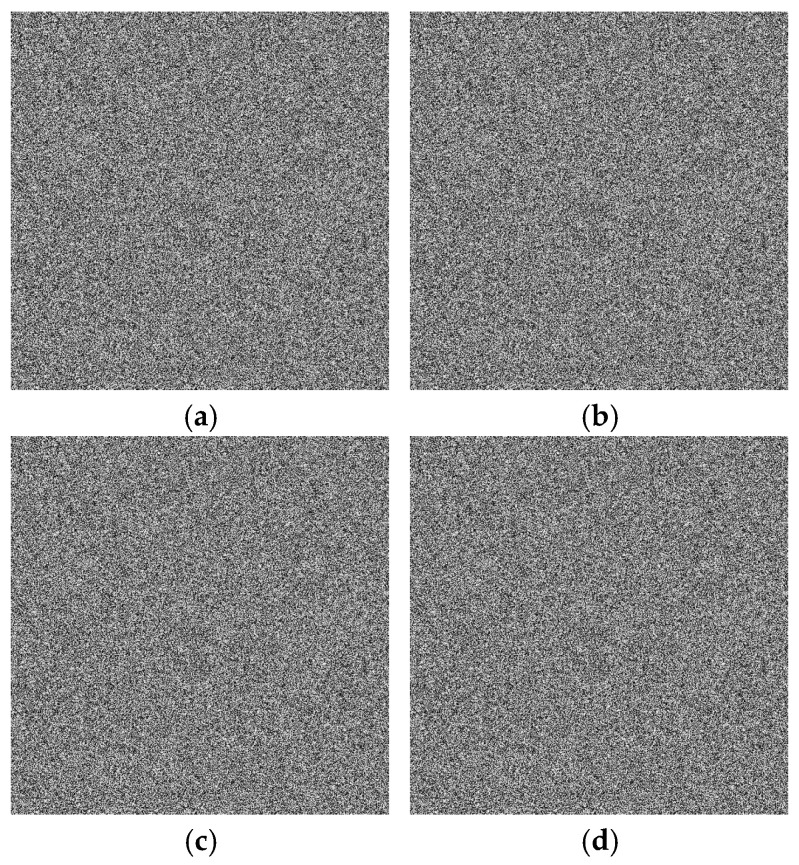
Key sensitivity analysis results: (**a**) decrypted image when a is slightly altered; (**b**) decrypted image when b is slightly altered; (**c**) decrypted image when $x_{0}$ is slightly altered; (**d**) decrypted image when $y_{0}$ is slightly altered.

Key sensitivity is critical for encryption algorithm security. A robust algorithm exhibits high key sensitivity, where even slight key alterations drastically change the encryption outcome. To validate this, we altered key components $\left(a,b,x_{0},y_{0}\right)$ by $10^{-15}$, then encrypted the same image using both original and altered keys. The encrypted images were compared using NPCR (Number of Pixels Change Rate) and UACI (average change intensity). As shown in Table 9, even a $10^{-15}$ key change results in NPCR near 99.6% and UACI near 33.5%. Figure 15a–d demonstrate that images encrypted with slightly altered keys are entirely different from the original, resembling random noise. This indicates the proposed algorithm’s high key sensitivity and resistance to differential attacks. Even with approximate keys, decryption fails.

### 5.6. Information Entropy

Information entropy is a critical metric for quantifying the randomness of information in an image, reflecting the uncertainty of pixel value distribution. For an encrypted image, a higher information entropy indicates greater randomness, thereby reducing the risk of leaking information about the original image. For grayscale images, information entropy is calculated using Shannon entropy. The corresponding formula is provided in Equation (30):

(30) $$H=-\sum_{i=0}^{255}P\left(i\right)\log_{2}P\left(i\right)$$

where P(i) represents the proportion of pixels with grayscale value i. For an 8-bit grayscale image, an ideal encrypted image should exhibit an information entropy value close to 8.

As shown in Table 10, after encryption using the proposed algorithm, the information entropy values of all ciphertext images increase significantly and are very close to the theoretical maximum of 8. Furthermore, these values are generally slightly higher than those reported in other recent comparative studies. This result clearly indicates that the proposed algorithm significantly enhances the randomness and uncertainty of the image’s pixel distribution, effectively eliminating the statistical regularities of the original image.

### 5.7. Interference Attack

The interference attack test is used to evaluate the robustness of an encryption algorithm when the encrypted image suffers damage (such as added noise or image cropping). An excellent encryption system should exhibit a certain degree of interference resistance, ensuring that the image can still be correctly decrypted or maintain its security after being subjected to interference. Experiments typically involve adding salt-and-pepper noise or occlusion blocks to the encrypted image, followed by decryption to observe whether the original image can be recovered with recognizable features. Two commonly used evaluation metrics are PSNR (Peak Signal-to-Noise Ratio) and SSIM (Structural Similarity Index).

PSNR quantifies the magnitude of pixel-level error between images; the smaller the error, the higher the PSNR, and the less severe the image distortion. Its calculation formulas are provided in Equations (31) and (32):

(31) $$PSNR=10\ln\left(\frac{{MAX}_{I}^{2}}{MSE}\right)$$

(32) $$MSE=\frac{1}{m\cdot n}\sum_{i=1}^{m}\sum_{j=1}^{n}{\left[I\left(i,j\right)-K\left(i,j\right)\right]}^{2}$$

where ${\mathrm{MAX}}_{I}$ denotes the maximum pixel value of the image (typically 255 for 8-bit images); MSE is the mean squared error; $I\left(i,j\right)$ represents the pixel of the original image; K(i,j) denotes the pixel of the decrypted (distorted) image; and m×n is the image size.

SSIM evaluates image similarity across three dimensions: structure, luminance, and contrast; a value closer to 1 indicates higher similarity. Its calculation formula is provided in Equation (33):

(33) $$SSIM\left(x,y\right)=\frac{\left(2\mu_{x}\mu_{y}+C_{1}\right)\left(2\sigma_{xy}+C_{2}\right)}{\left(\mu_{x}^{2}+\mu_{y}^{2}+C_{1}\right)\left(\sigma_{x}^{2}+\sigma_{y}^{2}+C_{2}\right)}$$

where $\mu_{x}$ and $\mu_{y}$ are the means of the image patches; $\sigma_{x}^{2}$ and $\sigma_{y}^{2}$ are the variances of the image patches; $\sigma_{xy}$ is the covariance of the image patches; and $C_{1}$ and $C_{2}$ are stabilizing constants to prevent division by zero (typically set as $C_{1}={\left(K_{1}L\right)}^{2}$, $C_{2}={\left(K_{2}L\right)}^{2}$, where L is the maximum pixel value, 255).

**Figure 16:**
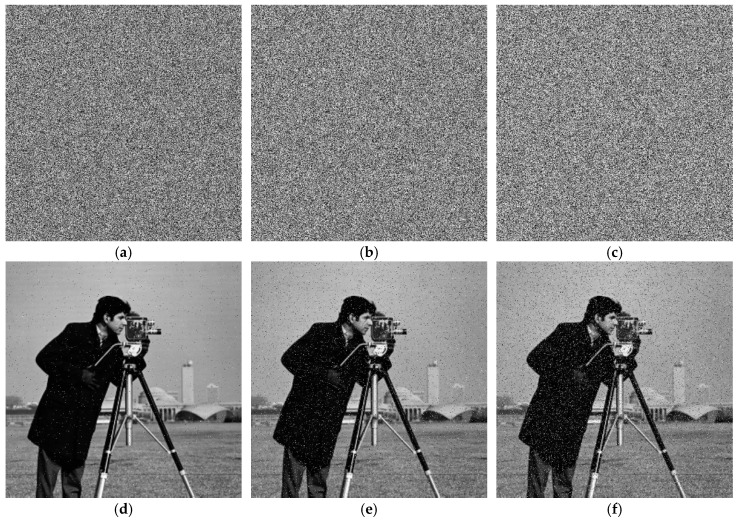
Salt-and-pepper noise analysis: (**a**–**c**) are ciphertext images that have been subjected to salt-and-pepper noise attacks with densities of 0.01, 0.05, and 0.1, respectively; (**d**–**f**) are the decrypted images of (**a**–**c**), respectively.

To evaluate the algorithm’s resistance to noise interference, we added salt-and-pepper noise (SPN)—a common impulse noise model—to ciphertext images at varying densities. The noise densities used in the experiments were 0.01, 0.05, and 0.1. The top row of Figure 16 shows the noisy ciphertext images (using Cameraman as an example) with SPN added at the respective densities, while the bottom row displays their corresponding decrypted results. Visual inspection reveals that although noise introduction visibly degrades the quality of decrypted images—particularly at high noise densities—the algorithm can still effectively recover the main features and content of the original image from the noisy ciphertext. The recovery effect is particularly pronounced at low noise densities.

We further calculated PSNR and SSIM metrics for quantitative analysis. For SPN densities of 0.01, 0.05, and 0.1, the PSNR values of the decrypted images were 28.2993 dB, 21.3929 dB, and 18.3711 dB, respectively; the corresponding SSIM values were 0.8326, 0.4576, and 0.2818. These quantitative results—especially the relatively high PSNR maintained at low noise density and the SSIM values reflecting the preservation of structural information—collectively confirm that the proposed 2D-ASM algorithm exhibits good resistance to salt-and-pepper noise attacks.

**Figure 17:**
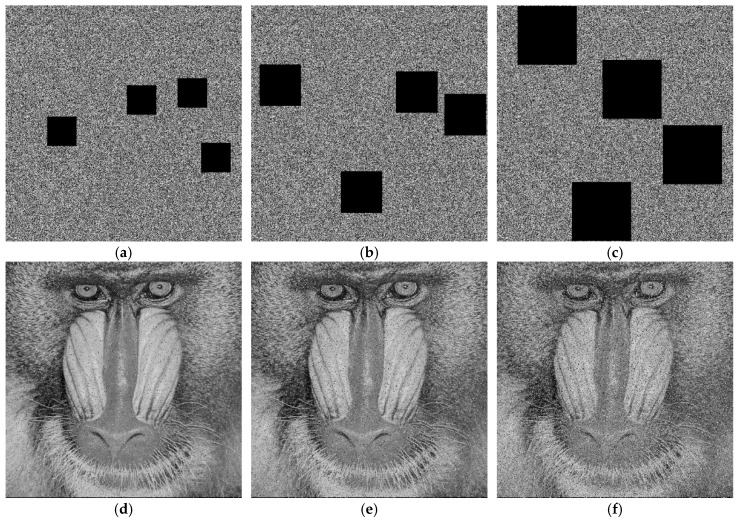
Cropping attack analysis: (**a**–**c**) are ciphertext images that have been subjected to cropping attacks with data loss rates of 1/16, 1/8, and 1/4, respectively; (**d**–**f**) are the decrypted images of (**a**–**c**), respectively.

For the cropping attack test, we divided the ciphertext image into four equal regions and randomly selected non-overlapping regions for occlusion (by setting pixels to zero or replacing them with specific values), simulating data loss ratios of 1/16, 1/8, and 1/4. The top row of Figure 17 shows the ciphertext images (using Baboon as an example) subjected to cropping attacks at different proportions, while the bottom row displays their corresponding decrypted images. From the decrypted results, it is evident that as the cropping ratio (i.e., the amount of data loss) increases, the quality of the decrypted image degrades, with increased blurriness or artifacts. However, even with data loss as high as 1/4, the decrypted image still retains the main contours and structural information of the original image, and its content remains partially recognizable.

For cropping ratios of 1/16, 1/8, and 1/4, the obtained PSNR values were 21.6041 dB, 18.6511 dB, and 15.0364 dB, respectively; the corresponding SSIM values were 0.6703, 0.5035, and 0.1636. These results—particularly the maintenance of relatively reasonable PSNR values even under significant cropping rates—indicate that the proposed encryption algorithm exhibits satisfactory robustness when facing substantial data loss.

### 5.8. Encryption and Decryption Speed Analysis

The computational complexity of the proposed ASM-IE scheme is analyzed with respect to an input image of size M × N. Let n = MN denote the total number of pixels. The encryption process mainly consists of chaotic sequence generation, dynamic DNA encoding, diffusion operations, and row-column scrambling. According to the analysis, the chaotic sequence generation has a complexity of O(6MN). The dynamic DNA encoding has a complexity of O (M + 4N) for the confusion stage. The diffusion operations have a complexity of O(12MN). Finally, based on the different complexities of the steps, the total complexity of the ASM-IE is O(12MN), indicating that the algorithm scales linearly with the image size. Although approximately a constant number of elementary arithmetic and logical operations (about 12 per pixel) are involved, the asymptotic growth remains linear. The actual execution time was measured using MATLAB R2021b on a PC equipped with an Intel i5 processor and 32 GB RAM. As shown in Table 11, the average encryption and decryption times for 512 × 512 test images are approximately 0.94 s and 0.30 s, respectively, which are significantly faster than ref. [17] and comparable to ref. [56]. These results demonstrate that the proposed ASM-IE scheme achieves favorable computational efficiency while maintaining strong security performance, making it practically feasible for real-time image encryption applications.

## 6. Conclusions

This paper presents a novel image encryption scheme that synergistically integrates the proposed two-dimensional Ackley-Sine chaotic map (2D-ASM) with dynamic DNA coding and operational rules, which is devised to mitigate the inherent drawbacks of conventional chaos-based image encryption methods in chaotic system performance and encryption mechanism design. The proposed 2D-ASM is constructed by incorporating the multimodal characteristics of the Ackley function with sine and cosine nonlinear transformation, and comprehensive chaotic characteristic analysis verifies that this map exhibits superior dynamical performance compared with state-of-the-art 2D chaotic systems, including an unbounded chaotic parameter range, ultra-high positive Lyapunov exponents (in excess of 23), Permutation Entropy approaching the ideal value of 1 across the full parameter domain, and full compliance with all 15 sub-tests of the NIST SP 800 randomness criterion. The encryption scheme takes the SHA-256 hash value of the plaintext image and four external keys as the joint input to generate the initial conditions and control parameters of the 2D-ASM system, which not only realizes the tight correlation between the encryption keystream and plaintext content, but also constructs an ultra-large key space of 2256 and achieves extreme key sensitivity—with the NPCR of the ciphertext image reaching approximately 99.6% even when the key is perturbed by 10–15. Through the cascaded implementation of pixel-level permutation, multi-layer diffusion and dynamic DNA-level operations, the scheme effectively eliminates the statistical characteristics and spatial correlation of plaintext images, generating ciphertext images with uniformly distributed histograms, adjacent pixel correlation coefficients close to 0, and information entropy approaching the theoretical upper limit of 8 for 8-bit grayscale images. The NPCR and UACI values of the ciphertext are stably maintained at approximately 99.6% and 33.4% respectively, which demonstrates that the scheme can effectively resist statistical attacks, differential attacks, salt-and-pepper noise attacks and cropping attacks. In terms of computational performance, the scheme has a linear computational complexity of O(12MN) for an M × N image, and the average encryption and decryption time for 512 × 512 standard test images is only about 0.94 s and 0.30 s respectively, realizing a favorable balance between encryption security and real-time performance, and meeting the application requirements of practical image encryption scenarios.

While the proposed ASM-IE scheme achieves excellent comprehensive performance in security and efficiency, several limitations still exist in its practical application: the robustness of the scheme degrades significantly under high-density noise interference and severe cropping attacks, the multi-step judgment and modular calculation in DNA-level operations lead to relatively high computational overhead for the module, and the performance verification of the scheme is currently limited to standard grayscale images with fixed resolution, with no in-depth exploration on special image types such as medical and remote sensing images and hardware implementation on FPGA/ASIC platforms. Future research will focus on four aspects to further optimize and expand the scheme: first, integrating error correction coding and adaptive denoising algorithms to enhance the anti-interference capability of the scheme under high-intensity attacks; second, simplifying the logical structure of DNA operations via lookup table design and parallel computing to reduce computational overhead; third, extending the scheme to multi-channel color image encryption and domain-specific image encryption scenarios, and completing the hardware implementation and optimization of the scheme for embedded security systems; fourth, introducing dynamic key update mechanisms and deep learning-based adaptive encryption strategies to enhance the resistance of the scheme to advanced cryptanalytic attacks such as differential fault analysis and algebraic attacks, and further exploring the integration of the scheme with blockchain and homomorphic encryption technologies to expand its application in secure cloud computing and distributed intelligent systems.

## Figures and Tables

**Table 1:** Lists of different 2D chaotic systems.

References	2D Chaotic System	Parameter
Cao et al. (2020) [7]	$\left\{\begin{array}{l} x_{i+1}=\sin\left(\frac{a}{y_{i}}\right)\sin\left(\frac{b}{x_{i}}\right),\\ y_{i+1}=\sin\left(\frac{a}{x_{i}}\right)\sin\left(\frac{b}{y_{i}}\right). \end{array}\right.$	a,b
Gao (2021) [8]	$\left\{\begin{array}{l} x_{i+1}=\sin\left(\frac{h\pi }{\sin\left(y_{i}\right)}\right),\\ y_{i+1}=\sin\left(\pi x_{i}y_{i}\right). \end{array}\right.$	h
Hua et al. (2021) [9]	$\left\{\begin{array}{l} x_{i+1}=\cos\left(4ax_{i}\left(1-x_{i}\right)+b\sin\left(\pi y_{i}\right)+1\right),\\ y_{i+1}=\sin\left(4ay_{i}\left(1-y_{i}\right)+b\sin\left(\pi x_{i}\right)+1\right). \end{array}\right.$	a,b
Teng et al. (2021) [10]	$\left\{\begin{array}{l} x_{i+1}=\sin\left(\frac{a}{\sin\left(y_{i}\right)}\right),\\ y_{i+1}=\beta \sin\left(\pi \left(x_{i}+y_{i}\right)\right). \end{array}\right.$	α,β
Nan et al. (2022) [11]	$\left\{\begin{array}{l} x_{i+1}=\cos\left(\pi^{2}\left(4\mu x_{i}\left(1-x_{i}\right)+py_{i}\left(1-y^{2}\right)\right)+\frac{\pi }{2}\right),\\ y_{i+1}=\cos\left(\pi^{2}\left(4\mu y_{i}\left(1-y_{i}\right)+px_{i}\left(1-x_{i+1}^{2}\right)\right)+\frac{\pi }{2}\right). \end{array}\right.$	μ,p
Zhu et al. (2022) [12]	$\left\{\begin{array}{l} x_{i+1}=x_{i}+\frac{h^{v}}{\Gamma \left(1+v\right)}\cos\left(\frac{2\pi x_{i}}{2\mu x_{i}^{4}}-y_{i}\right),\\ y_{i+1}=y_{i}+\frac{h^{v}}{\Gamma \left(1+v\right)}\cos\left(\mu \pi \left(x_{i}+y_{i}\right)\right). \end{array}\right.$	h,v,μ
Hu et al. (2023) [13]	$\left\{\begin{array}{l} x_{i+1}=\left(ax_{i}+cy_{i}^{\gamma }\right)\mathrm{mod}\beta ,\\ y_{i+1}=by_{i}\mathrm{mod}\beta . \end{array}\right.$	a,b,c,γ
Du et al. (2024) [16]	$\left\{\begin{array}{l} x_{i+1}=\mu \sin\left(\pi \left(4\lambda_{1}y_{i}\left(1-y_{i}\right)+\gamma_{1}x_{i}^{2}\right)\right),\\ y_{i+1}=\beta \left(4\lambda_{2}x_{i}\left(1-x_{i}\right)+\gamma_{2}y_{i}^{2}-{\left(4\lambda_{2}x_{i}\left(1-x_{i}\right)+\gamma_{2}y_{i}^{2}\right)}^{3}\right). \end{array}\right.$	$\begin{array}{l} \mu ,\beta ,\lambda_{1},\\ \lambda_{2},\gamma_{1},\gamma_{2} \end{array}$
Zhang et al. (2025) [17]	$\left\{\begin{array}{l} x_{i+1}=\sin^{2}\left(m\pi^{2}\left(\ln\left(x_{i}\right)\exp\left(y_{i}\right)+\ln\left(y_{i}\right)\exp\left(x_{i}\right)\right)\right),\\ y_{i+1}=\sin^{2}\left(n\pi^{2}\left(\ln\left(x_{i}y_{i}\right)\exp\left(x_{i}y_{i}\right)\right)\right). \end{array}\right.$	m,n

**Table 2:** NIST 800 test results.

No.	Sub-Tests	*p*-Value	Result
		≥0.01	
01	Frequency	0.94738	pass
02	Frequency within Block	0.67557	pass
03	Runs	0.59196	pass
04	Longest Run	0.48327	pass
05	Rank	0.03247	pass
06	Fourier Transform	0.1916	pass
07	Non-Overlapping Template	0.12493	pass
08	Overlapping Template	0.11204	pass
09	Universal Statistical	0.69932	pass
10	Linear Complexity	0.50821	pass
11	Serial *p*-value 1	0.51297	pass
	Serial *p*-value 2	0.26037	pass
12	Approximate Entropy	0.44857	pass
13	Cumulated Sum (F)	0.98891	pass
	Cumulated Sum (R)	0.70359	pass
14	Random Excursion	0.40928	pass
15	Random Excursion Variant	0.30404	pass

**Table 3:** DNA coding operation rules.

Rules	1	2	3	4	5	6	7	8
00	T	T	A	A	C	C	G	G
01	G	C	G	C	A	T	A	T
10	C	G	C	G	T	A	T	A
11	A	A	T	T	G	G	C	C

**Table 4:** DNA algebraic XOR rule.

⊕ (XOR)	T	A	C	G
T	A	T	G	C
A	T	A	C	G
C	C	G	T	A
G	G	C	A	T

**Table 5:** DNA algebraic addition rule.

+(ADD)	T	A	C	G
T	C	T	G	A
A	T	A	C	G
C	G	C	A	T
G	A	G	T	C

**Table 6:** DNA algebraic subtraction rule.

−(SUB)	T	A	C	G
T	A	T	G	C
A	G	A	C	T
C	T	C	A	G
G	C	G	T	A

**Table 7:** Comparison results of adjacent pixel correlation.

Method	Image	Plain Image			Cipher Image		
		Horizontal	Vertical	Diagonal	Horizontal	Vertical	Diagonal
The current study	Baboon	0.7670	0.7258	0.8694	0.0020	0.0008	−0.0006
	Barbara	0.9615	0.8629	0.8433	−0.0031	0.0059	0.0027
	Goldhill	0.9737	0.9706	0.9545	0.0049	0.0064	−0.0011
	Lena	0.9852	0.9682	0.9593	−0.0062	−0.0049	0.0029
	Peppers	0.9822	0.9788	0.9678	−0.0026	−0.0076	−0.0020
	Cameraman	0.9898	0.9823	0.9743	0.0071	0.0045	−0.0003
Gao (2021) [8]	5.1.09	0.9388	0.9006	0.9050	−0.0054	−0.0017	−0.0021
	5.1.10	0.8681	0.9043	0.8313	−0.0024	−0.0069	0.0007
	5.1.11	0.9521	0.9532	0.9082	0.0010	−0.0026	0.0017
	5.1.12	0.9742	0.9560	0.9399	0.0076	−0.0118	−0.0062
	5.1.13	0.8696	0.8727	0.7539	0.0027	0.0005	−0.0004
	5.1.14	0.8982	0.9461	0.8522	−0.0065	0.0036	−0.0050
Lai et al. (2023) [18]	Lena	0.9542	0.8831	0.9205	−0.0021	−0.0012	0.0017
	Baboon	0.7889	0.6844	0.6790	0.0015	0.0048	0.0016
	Barbara	0.9210	0.9109	0.8478	0.0010	−0.0011	−0.0012
	Peppers	0.9620	0.9510	0.9248	0.0005	0.0004	0.0032
	Boats	0.9285	0.8688	0.8844	0.0023	0.0032	0.0016
	Airplane	0.9064	0.8209	0.8402	0.0038	−0.0048	−0.0001

**Table 8:** Comparation of NPCR and UACI.

Method	Image	NPCR/%	UACI/%
The current study	Baboon	99.6101	33.4399
	Barbara	99.6177	33.4633
	Goldhill	99.6056	33.4377
	Lena	99.5995	33.4583
	Peppers	99.6017	33.4238
	Cameraman	99.6192	33.4384
Zhu et al. (2022) [12]	5.1.09	99.5700	33.4300
	5.1.10	99.5700	33.4400
	5.1.11	99.5600	33.4600
	5.1.12	99.5800	33.4800
	5.1.13	99.5700	33.4700
	5.1.14	99.5500	33.5200
Lai et al. (2023) [18]	Lena	99.6036	33.4523
	Baboon	99.6025	33.4494
	Barbara	99.6109	33.4547
	Peppers	99.6009	33.4564
	Boats	99.6086	33.4551
	Airplane	99.5948	33.4790

**Table 9:** NPCR and UACI with slightly altered decryption key.

Key	$a+10^{-15}$	$b+10^{-15}$	$x_{0}+10^{-15}$	$y_{0}+10^{-15}$
NPCR	99.6124%	99.5956%	99.5975%	99.5914%
UACI	33.5116%	33.3952%	33.4913%	33.4506%

**Table 10:** Comparation of information entropy.

Method	Image	Ciphertext	Plaintext
The current study	Baboon	7.9993	7.5379
	Barbara	7.9994	7.4664
	Goldhill	7.9994	7.4778
	Lena	7.9994	7.4456
	Peppers	7.9993	7. 5715
	Cameraman	7.9991	7.0480
Zhu et al. (2022) [12]	Lena	7.9973	7.4464
	Brain	7.9974	4.6652
	Woman	7.9971	7.2695
	Peppers	7.9973	7.5715
	Barbara	7.9968	7.5252
Lai et al. (2023) [18]	Lena	7.9975	
	Baboon	7.9970	
	Barbara	7.9970	
	Peppers	7.9965	
	Boats	7.9968	
	Airplane	7.9967	

**Table 11:** Encryption and Decryption Times for 512 × 512 Images.

Original Image	Encryption Time			Decryption Time		
	ASM-IE	Ref. [17]	Ref. [56]	ASM-IE	Ref. [17]	Ref. [56]
Lena	0.9463	3.7066	0.9674	0.3032	3.8762	0.6993
Baboon	0.9295	3.7147	0.9748	0.3034	3.6438	0.6641
Peppers	0.9491	3.7129	0.9636	0.2998	3.6275	0.6670
Cameraman	0.9399	3.7800	0.9674	0.2999	3.6830	0.6452

## Data Availability

Data will be made available on request.
